# *In vivo* confocal microscopy in goldenhar syndrome: a case report

**DOI:** 10.1186/1471-2415-13-55

**Published:** 2013-10-16

**Authors:** Giacinto Triolo, Giulio Ferrari, Claudio Doglioni, Paolo Rama

**Affiliations:** 1 Department of Ophthalmology, San Raffaele Scientific Institute, Milan, Italy; 2 Cornea and Ocular Surface Unit, San Raffaele Scientific Institute, Milan, Italy; 3 Pathology Unit, San Raffaele Scientific Institute, Milan, Italy; 4 Università Vita-Salute San Raffaele, Milan, Italy

**Keywords:** *In vivo* confocal microscopy, Goldenhar syndrome, Dermoid

## Abstract

**Background:**

Goldenhar Syndrome is characterized by malformations of multiple anatomical districts. Between these, bulbar dermoids are common and represent a significant clinical problem as they can affect both ocular function and aesthetic comfort.

Histologic characterization of dermoids has been extensively performed; however, no reports exist describing *in vivo* confocal microscopy (IVCM) of these lesions. We aimed to (i) describe the *in vivo* confocal morphology of limbal dermoids in Goldenhar syndrome and (ii) compare these findings with standard light microscopy.

**Case presentation:**

A 15-year-old Caucasian female affected by Goldenhar Syndrome showed a left, infero-temporal, limbal neoformation, with extension to the left orbital region. Prior to surgical removal, IVCM was performed with the Heidelberg Retina Tomograph II, Cornea Module, using the “section” modality. The IVCM sections showed structures resembling corneal epithelium and vascular structures. Surgical removal of the lesion was decided as it caused poor eyelid closure. After surgical removal, sectioning and standard optical microscopy were performed. The comparison between IVCM imaging and standard microscopy sections were highly correlated in the detection of the pilar and vascular structures.

**Conclusions:**

This study showed that IVCM may be a useful technique to study limbal dermoids, given its ability to detect typical microscopic features and its comparability to optical microscopy, which is the current standard.

## Background

Goldenhar Syndrome (GS) is a polymalformation syndrome characterized by uni- or bilateral microtia, mandibular region hypoplasia, hemifacial microsomy, ocular and auricolar anomalies, skeleton vertebral anomalies and other types of malformations [1,2]. GS has a variable frequency from 1:3500 to 1:26000 born children with a male predominance (M:F=3:2). The majority of GS cases are sporadic, but familial cases support an autosomal dominant or recessive pattern of inheritance [3,4].

The etiology is still unknown and debated. Several pathogenetic mechanisms have been proposed; however it is commonly accepted that some type of vascular perturbation and neural crestopathy occur in a critical time point of ocular embryogenesis [5].

Ocular findings are frequently reported in GS: epibulbar dermoids are the most common manifestation, reported in a variable percentage of these patients (6-39%) [1,2,6]; other ocular features include microphthalmia (6%), cataract, chorioretinal atrophy, nasolacrimal duct stenosis and coloboma of eyelids (3%) [1]. Optical microscopy studies demonstrated a dense fibrous derma-like tissue, with the presence of sporadic hair follicles; a smaller, deeper component was also described, consisting of hypodermic tissue with diffuse adipocytes [7,8].

The use of *in vivo* confocal microscopy (IVCM) has already entered the clinical practice for the diagnosis of amoebic and fungal keratitis, and in the follow-up of refractive surgery patients [9].

However, the histopathology of the epibulbar dermoids has not been studied with IVCM yet.

We report the case of a 15 year old female patient with diagnosis of GS: she underwent surgical excision of left, infero-temporal limbal dermoid. The purpose of this study is to describe the pathology of limbal dermoids as imaged with IVCM, and to compare these findings with standard optical microscopy.

## Case presentation

**Figure 1:**
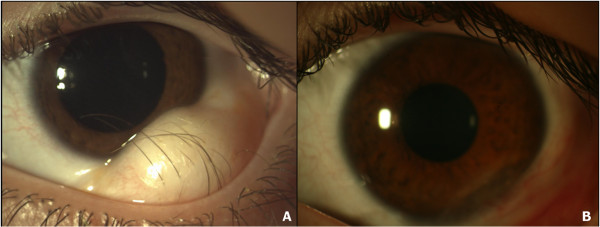
**Clinical picture of the left eye.** Note the infero-temporal, limbal dermoid, 15x15 mm in diameter, with extention to the left orbital region, before (panel **A**) and after (panel **B**) its surgical removal. Note the pilar structures originating from the lesion.

A 15 year old patient with diagnosis of Goldenhar Syndrome came to our attention for the evaluation of a left, infero-temporal, limbal neoformation, 15×15 mm in diameter, with extension to the left orbital region, causing incomplete occlusion of the left eyelid (Figure 1, panel A).

The clinical ocular findings were normal for the rest, with a BCVA of 0.1 logMAR, transparent central cornea and normal features of the anterior chamber. As the lesion did not allow normal closure of the eyelids and there was an aesthetic issue, surgical removal of the lesion was decided. The post surgical course was regular with no complications (Figure 1, panel B).

**Figure 2:**
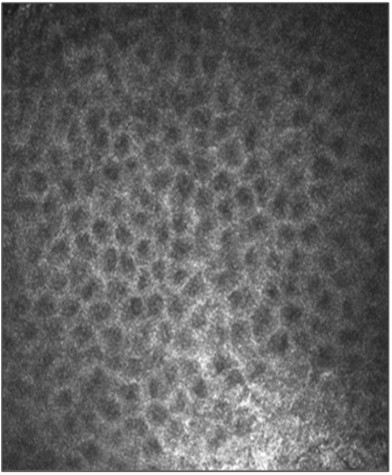
**Confocal image of the lesion (18 μm of depth).** It shows dermoid epithelium. Note the regular pattern of dark gray roundish structures, each surrounded by a thin whitish ring.

Prior to surgical removal, *in vivo* confocal microscopy (IVCM) was performed with the Heidelberg Retina Tomograph II, Cornea Module, which allows *in vivo* histological imaging of sections at different depths parallel to the scanned surface. A 400 μm field of view lens was used. The most superficial *in vivo* confocal sections (18 μm of depth) showed a highly regular pattern characterized by dark gray roundish structures, each surrounded by a thin whitish ring, which may represent corneal epithelium (Figure 2).

**Figure 3:**
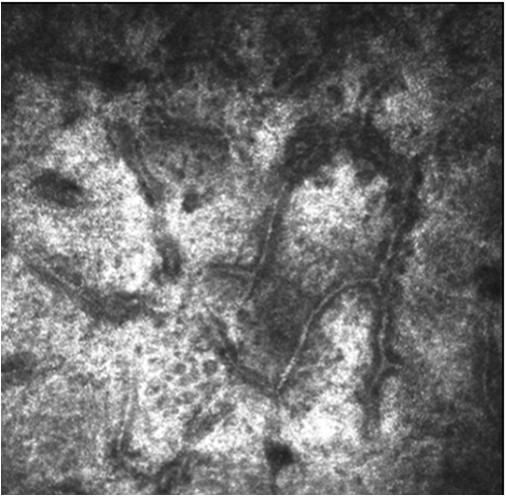
**Confocal image of the lesion (63 μm of depth).** It shows the longitudinal course of continuous, branched, tubular structures, probably of vascular nature.

Deeper confocal sections, taken at 63 μm, showed the longitudinal course of continuous, branched, tubular structures, probably of vascular nature (Figure 3). These structures could have been squeezed on the same plane by the presence of compressive phenomena within the lesion, such as the formation of a hair bulb and/or the growth of the hair itself.

**Figure 4:**
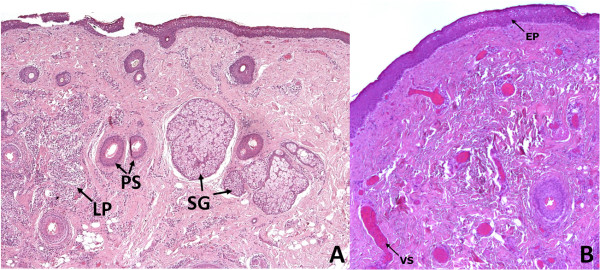
**Histologic sagittal section of the dermoid lesion.** It shows stratified squamous epithelium (EP) with irregular, thin keratinized superficial layer, disorganized pilar stuctures (PS), sebaceous glands (SG), lymphoplasmacytic inflammatory infiltrate (LP) (panel **A**) and vascular structures (VS) (panel **B**) (hematoxylin and eosin, × 50).

After removal, the tissue was oriented, fixed in 10% formalin and processed to obtain standard microscopy sections perpendicular to the ocular surface. This confirmed the lesion to be a solid limbal dermoid associated with Goldenhar Syndrome. The tissue was characterized by stratified squamous epithelium resembling epidermis with an irregularly thin, keratinized superficial layer (Figure 4, panel A and B). The stroma appeared dense and paucicellular and contained a disordered number of pilar structures, sebaceous glands, lymphoplasmacytic inflammatory infiltrate (Figure 4, panel A), and vascular structures (Figure 4, panel B). Empty vacuolar structures were also identified, representing adipose tissue (Figure 4).

**Figure 5:**
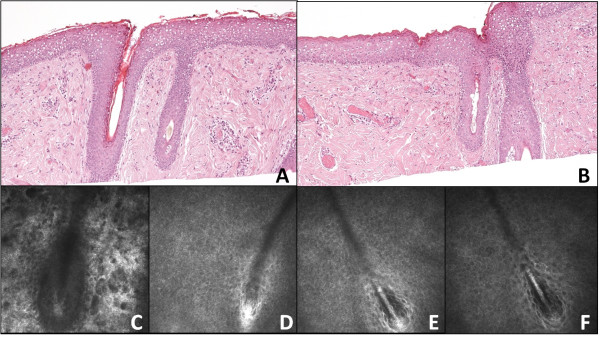
**Pilar structures: comparison between confocal and histologic microscopy.** Histologic coronal sections (panels **A**, **B**; × 100) with the corresponding confocal images (panels **C**, **D**, **E**, **F**) highlighting pilar structures.

**Figure 6:**
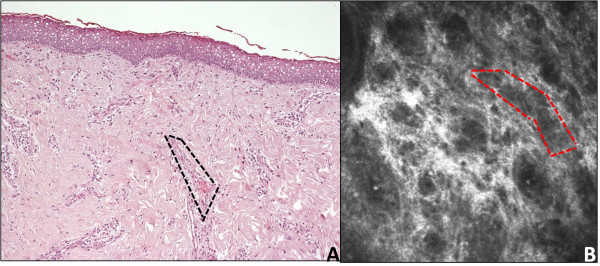
**Vascular structures: comparison between confocal and histologic microscopy.** The confocal image (panel **A**) displays a quite regular dark-gray microareolar pattern in the context of the connective tissue stroma (red square), probably corresponding to the vascular structures seen in corresponding histologic plane of section (panel **B**, black square; × 100).

Although obvious differences associated with the two different techniques, we strived to reduce these to a minimum. To this end, the pathologist prepared sections parallel to the surface of the lesion, rather than perpendicular to it. He provided histological sections at the same depth as optical sections obtained with IVCM. Although we cannot be absolutely sure of co-localization of the 2 structures, the anatomical sample was oriented at the time of removal to facilitate sectioning up to a depth of about 70 μm. Given the comparable depth at which the dermoid tissue may be investigated by IVCM, this procedure has allowed to achieve histological sections overlapping to IVCM images. Optical microscopy and IVCM were clearly comparable in the detection of pilar structures (Figure 5). Finally, a quite regular dark-gray microareolar pattern mixed with the connective tissue stroma was observed (Figure 6, panel A - red square), possibly corresponding to the vascular structures seen in the overlapping histological section plane (Figure 6, panel B – black square).

## Conclusions

This study showed that IVCM may be a valid technique to characterize the histology of limbal dermoids, given its ability to recognize the main typical microscopic features of this lesion and its comparability to optical microscopy, which is the current standard. Specifically, both methods were highly comparable in the detection of pilar and vascular structures.

IVCM also has intrinsic limitations, such as the impossibility to perform specific staining. Moreover, it is difficult to image exactly the same area with both IVCM and optical microscopy, due to the absence of unequivocal reference points. However, we tried to reduce these downsides by careful orienting the dermoid when preparing histologic sections. In fact, optical microscopy for ocular pathology specimens commonly performed perpendicularly to the surface of the lesion, differently from IVCM, which captures sections parallel to the surface. For this reason we specifically asked the pathologist to obtain histologic sections corresponding to the confocal imaging.

In light of our results, we plan to increase the patient sample to further confirm our findings in a group of patients affected with Goldenhar syndrome. In particular, it would be interesting to test whether peculiar histologic phenotypes (i.e. extensive vascular ramification, microareolar pattern, density of pilar structures) are associated with a different growth rate of these lesions. In these regards, IVCM could represent a helpful prognostic tool to predict the evolution of the lesion when discussing surgical removal of dermoids in Goldenhar Syndrome. In fact, given the extremely low rate of malignant transformation, “wait and see” could be a wise choice when they do not represent an aesthetic or functional issue. Moreover, this technique could play a key role in surgical planning, allowing prompt removal of lesions showing a “fast growing” phenotype, finally allowing better functional recovery.

## Consent

Written informed consent was obtained from the patient’s parents for publication of this case report and any accompanying images. A copy of the written consent is available for review by the Editor of this Journal.

## Competing interests

All authors declare that they have no competing interests.

## Authors’ contributions

GF acquired pre- and post-surgical clinical images, performed the *in vivo* confocal microscopy and critically reviewed the manuscript. GT has acquired the informed consent of the patient, collected the clinical and histologic imaging, drafted the manuscript and prepared the images. CD performed the histological study, confirming the diagnosis of limbal dermoid, and interpreted the images of optic and *in vivo* confocal microscopy. PR visited the patient and laid the clinical suspicion of limbal dermoid associated with Goldenhar syndrome, surgically removed the lesion and followed the patient for follow-up visits after the surgery. All authors read and approved the final manuscript.

## Pre-publication history

The pre-publication history for this paper can be accessed here:

http://www.biomedcentral.com/1471-2415/13/55/prepub
